# Development and validation of a measurement scale to assess nursing students’ readiness for the flipped classroom in Sri Lanka

**DOI:** 10.3352/jeehp.2020.17.41

**Published:** 2020-12-14

**Authors:** Punithalingam Youhasan, Yan Chen, Mataroria Lyndon, Marcus Alexander Henning

**Affiliations:** 1 Centre for Medical and Health Science Education, Faculty of Medical and Health Sciences, The University of Auckland, Auckland, New Zealand; 2 Department of Medical Education & Research, Faculty of Health-Care Sciences, Eastern University Sri Lanka, Batticaloa, Sri Lanka; Hallym University, Korea

**Keywords:** Nursing education, Psychometrics, Reproducibility of results, Statistical factor analysis, Sri Lanka

## Abstract

**Purpose:**

The aim of this study was to develop and validate a scale to measure nursing students’ readiness for the flipped classroom in Sri Lanka.

**Methods:**

A literature review provided the theoretical framework for developing the Nursing Students’ Readiness for Flipped Classroom (NSR-FC) questionnaire. Five content experts evaluated the NSR-FC, and content validity indices (CVI) were calculated. Cross-sectional surveys among 355 undergraduate nursing students from 3 state universities in Sri Lanka were carried out to assess the psychometric properties of the NSR-FC. Principal component analysis (PCA, n=265), internal consistency (using the Cronbach α coefficient, n=265), and confirmatory factor analysis (CFA, n=90) were done to test construct validity and reliability.

**Results:**

Thirty-seven items were included in the NSR-FC for content validation, resulting in an average scale CVI of 0.94. Two items received item level CVI of less than 0.78. The factor structures of the 35 items were explored through PCA with orthogonal factor rotation, culminating in the identification of 5 factors. These factors were classified as technological readiness, environmental readiness, personal readiness, pedagogical readiness, and interpersonal readiness. The NSR-FC also showed an overall acceptable level of internal consistency (Cronbach α=0.9). CFA verified a 4-factor model (excluding the interpersonal readiness factor) and 20 items that achieved acceptable fit (standardized root mean square residual=0.08, root mean square error of approximation=0.08, comparative fit index=0.87, and χ^2^/degrees of freedom=1.57).

**Conclusion:**

The NSR-FC, as a 4-factor model, is an acceptable measurement scale for assessing nursing students’ readiness for the flipped classroom in terms of its construct validity and reliability.

## Introduction

### Background/rationale

Contemporary nursing education aims to prioritize student-centred learning, which is perceived as high-order, flexible, and individualized [1]. Recent advances in technology have accelerated educational innovations such as blended learning by providing easy access to information [2,3]. Blended learning is a novel student-centred pedagogical approach that includes technology-mediated online and face-to-face (F2F) learning [2]. The flipped classroom (FC) is one of several modern blended learning strategies [3]. In FC, teachers use technology to share pre-class learning material to activate low-order learning, and students study the material before attending the F2F classroom. Teachers design the F2F classroom as an interactive educational environment by using student-centred teaching strategies that allow the students to apply or evaluate the learnt concepts [1,2].

The FC has entered into use in undergraduate nursing education and has been in the spotlight in discussions of the implementation of nursing curricula [2,3]. With an increasing emphasis on the FC, the available empirical evidence on the usage and efficacy of the FC in nursing education mainly refers to its effects on students’ academic achievements and does not take into account other aspects of educational effectiveness [1,2]. Thorndike [4] in 1932 outlined a law of learning according to which students’ readiness to learn is an indispensable factor for measuring the degree of success of academic achievement. Readiness for the FC can be conceived of as a concept describing the ability of an individual to benefit from blended learning [5]. Moreover, readiness refers to a level of mental and physical preparedness among learners when taking part in the FC [6]. Assessing students’ readiness is a preliminary step for implementing the FC [3]. However, there is limited research investigating students’ readiness for the FC educational process in the context of nursing education. Therefore, it would be valuable for educational and research purposes to develop a measurement instrument to investigate students’ readiness for the FC.

### Objectives

The aim of this study was to develop and validate a tool to measure nursing students’ readiness for the FC, namely the Nursing Students’ Readiness for Flipped Classroom (NSR-FC). Content validity, construct validity, and reliability tests were done to validate the measurement scale.

## Methods

### Ethics statement

The study was approved by the University of Auckland Human Participants Ethics Committee (reference no., 024079). Participants were provided with an information sheet before the anonymized questionnaire was administered. Participants were clearly informed that the voluntary return of the questionnaire to the collection box indicated their consent to participate in the anonymized survey.

### Study design

This was a psychometric study to validate the measurement scale based on experts’ opinion and survey results for the scale.

### Participants

Five experts participated in validity testing of the scale, and 265 undergraduate nursing students (in the 1st and 2nd academic years) from 3 state universities (Colombo University [n=141], University of Peradeniya [n=73], and Eastern University, Sri Lanka [n=51]) in Sri Lanka participated in exploratory factor analysis (EFA). Responses from 90 different undergraduate nursing students (3rd and 4th academic years) (University of Peradeniya [n=42] and Eastern University, Sri Lanka [n=48]) were employed for confirmatory factor analysis (CFA).

### Setting

This cross-sectional study was conducted in 2 steps. The first step involved the development of the NSR-FC. The second step was to investigate the psychometric properties of the NSR-FC.

### Step 1: Developing a scale for measuring nursing students’ readiness for the flipped classroom

The NSR-FC was drafted after reviewing the literature pertaining to learner readiness. The following existing inventories were used to generate items for the NSR-FC: E-Learning Readiness [7]; Online Learning Readiness Scale [6], and the ICT literacy scale [8]. However, none of the existing inventories had been developed specifically for nursing education. Therefore, 18 items (Q1–3, Q5–6, Q15–17, Q21–24, Q26, Q28–29, and Q33–35) were generated specifically to assess FC readiness by our research team based on our experience in teaching and learning in clinical and nursing education. Furthermore, 19 items were modified from the existing inventories and included in the NSR-FC. As a result, 37 preliminary items were included in the NSR-FC. A 5-point Likert scale was used, with responses ranging from 1 (strongly disagree) to 5 (strongly agree) as scale response anchors (Supplement 1).

### Step 2: Exploring the psychometric properties of the NSR-FC

#### Content validation of the NSR-FC

Content validation was done to assess the level of representativeness, relevance, understandability, and completeness of the NSR-FC. Five Sri Lankan content experts including 3 senior academics in health profession education and 2 academics in nursing participated and individually evaluated the degree of item significance for nursing students’ readiness to engage in the FC. The content validity indices (CVI) at the average scale-level (S-CVI/average) and item-level (I-CVI) were calculated using descriptive statistics. The I-CVI for each item on the NSR-FC was computed as the number of experts giving a rating of 1 (not relevant) to 4 (highly relevant) divided by the total number of experts who responded to the item. An I-CVI score of 0.78 or higher was considered to be adequate [9]. The S-CVI/average was calculated as the sum of the I-CVIs divided by the total number of items. An S-CVI/average score of 0.90 or above was considered as acceptable [9].

#### Construct validation of the NSR-FC

EFA and CFA were conducted for the investigation of construct validity of the NSR-FC. In total, 265 undergraduate nursing students participated in EFA. The Kaiser–Meyer–Olkin (KMO) test and the Bartlett test of sphericity were used to test the adequacy of the study’s sample and the suitability of orthogonal factor rotation [10]. Principal component analysis (PCA) was used with varimax rotation to identify the factors of the NSR-FC items that related to the corresponding variables. A parallel analysis was performed to confirm the extracted factors by comparing eigenvalues obtained from the raw data sets and randomly generated parallel dataset [11]. The factors that received eigenvalues higher than those from the corresponding datasets were included for further analysis [11]. The cut-off extraction value of factor loading was determined as 0.4 or above [12].

The degree of model fit was assessed through CFA, which utilized responses from 90 different undergraduate nursing students (Dataset 2). Four goodness-of-fit indices were calculated to estimate the global fit of the NSR-FC: the comparative fit index (CFI, with a threshold of >0.90); the root mean square error of approximation (RMSEA; a value of <0.05 was considered to indicate close fit, and a value between 0.05–0.08 to indicate reasonable fit); standardized root mean square residual (SRMR, with a threshold ≤0.08); and the chi-square statistic and its ratio to degrees of freedom (χ^2^/degrees of freedom [df], with a threshold of <5) [6,13].

#### Internal consistency of the NSR-FC

Internal consistency is commonly used to indicate the degree of reliability of a self-reporting questionnaire. The internal consistency of the NSR-FC was measured by computing the Cronbach α coefficient. A Cronbach α of 0.70 or higher was considered to indicate acceptable internal consistency, meaning that the observed score variance is reliable when compared with the true score variance [14].

### Study size

In accordance with the recommendation made by Lynn [15], 5 content experts were recruited for content validation. In total, 365 students participated in the study. Cattell [16] in 1978 recommended 3 to 6 samples per variable when conducting EFA. Therefore, we randomly selected students in their 1st and 2nd academic years ([35*6]<[n=265]) for EFA and the reliability test. The remaining sample (n=90) was used for CFA, which was sufficient to produce good agreement between the sample and population solutions (K value=0.92) [17].

### Statistical methods

Descriptive statistics were employed to calculate CVI measures using Microsoft Excel (Microsoft Corp., Redmond, WA, USA). PCA was applied using IBM SPSS ver. 26.0 (IBM Corp., Armonk, NY, USA) to explore the factor structure. Cronbach α coefficients were computed using IBM SPSS ver. 26.0 (IBM Corp.). The CFA goodness-of-fit indices were calculated using AMOS ver. 26.0 (IBM Corp.) [8,10].

## Results

### Content validation

At the item level, 91.9% of the NSR-FC’s items (n=34) had an I-CVI greater than or equal to 0.90. Two items of the NSR-FC (E1 & E2) received an I-CVI less than 0.78, namely “I can discipline myself to follow flipped learning” (I-CVI=0.75) and “I am committed to using flipped learning” (I-CVI=0.45). In addition, reviewers reported that E1 and E2 duplicated existing items, so these 2 items were excluded from the questionnaire. The S-CVI/average of the questionnaire achieved an acceptable level of 0.94 (Supplement 1).

### Exploratory factor analysis of the NSR-FC

The NSR-FC was explored using PCA to determine the optimal model that best represented the data. The KMO value of the NSR-FC was 0.873, suggesting that the samples were adequate for PCA. The Bartlett test of sphericity further affirmed the suitability of the data for PCA with orthogonal factor rotation (χ^2^=4,717.18, P<0.001). The PCA revealed that NSR-FC could be reduced to 5 factors, namely technological readiness, environmental readiness, personal readiness, pedagogical readiness, and interpersonal readiness. Parallel analysis confirmed the 5 factors in the NSR-FC. The percentage of variance explained by the rotated factor matrices ranged from 4.29% to 25.42% per factor, with the 5 factors explaining 55.25% of the overall variance. Factor loading after the rotation of each item is shown in (Table 1, Dataset 1).

Accordingly, technological readiness (factor 1) included 12 items (Q6–16 and Q18). The factor had a cumulative eigenvalue of 8.89 and accounted for 25.42% of the total variance. Environmental readiness (factor 2) comprised 5 items (Q20–21 and Q23–25), had a cumulative eigenvalue of 4.38, and accounted for 12.51% of the total variance. Personal readiness (factor 3) contained 6 items (Q1–4, Q17, and Q19). The factor had a cumulative eigenvalue of 2.81, and accounted for 8.02% of the total variance. Pedagogical readiness (factor 4) encompassed 7 items (Q22, Q26–29, and Q34–35), had a cumulative eigenvalue of 1.75, and accounted for 5.01% of the total variance. Interpersonal readiness (factor 5) included 4 items (Q30–33) and had a cumulative eigenvalue of 1.50, accounting for 4.29% of the total variance. Item Q5 obtained a factor extraction value of 0.25, which was deemed to be low; this item was therefore excluded from the NSR-FC.

### Internal consistency of the NSR-FC

The Cronbach α values of the NSR-FC’s factors ranged from 0.76 to 0.93, and the Cronbach α for the scale as a whole was 0.90, indicating excellent reliability. The intraclass correlation coefficients (ICCs) for the NSR-FC’s factors showed satisfactory results for each subscale (ICCs ranging from 0.76 to 0.92; P<0.01), and the overall ICC was 0.90, exhibiting an acceptable level of reliability. The corresponding data are presented in (Table 2, Dataset 1).

### Confirmatory factor analysis of the NSR-FC

Model 1 of the CFA denotes the baseline model, with 34 items as identified by PCA. In reference to model 1, the goodness-of-fit indices did not achieve an acceptable level, except for χ^2^/df. Therefore, model 2 was created by excluding 5 items (Q7, 10, 16, 22, and 33) that showed loading values less than 0.4; however, the goodness-of-fit values of model 2 were still below the cut-off level (Table 3, Dataset 2).

**Fig. 1.**
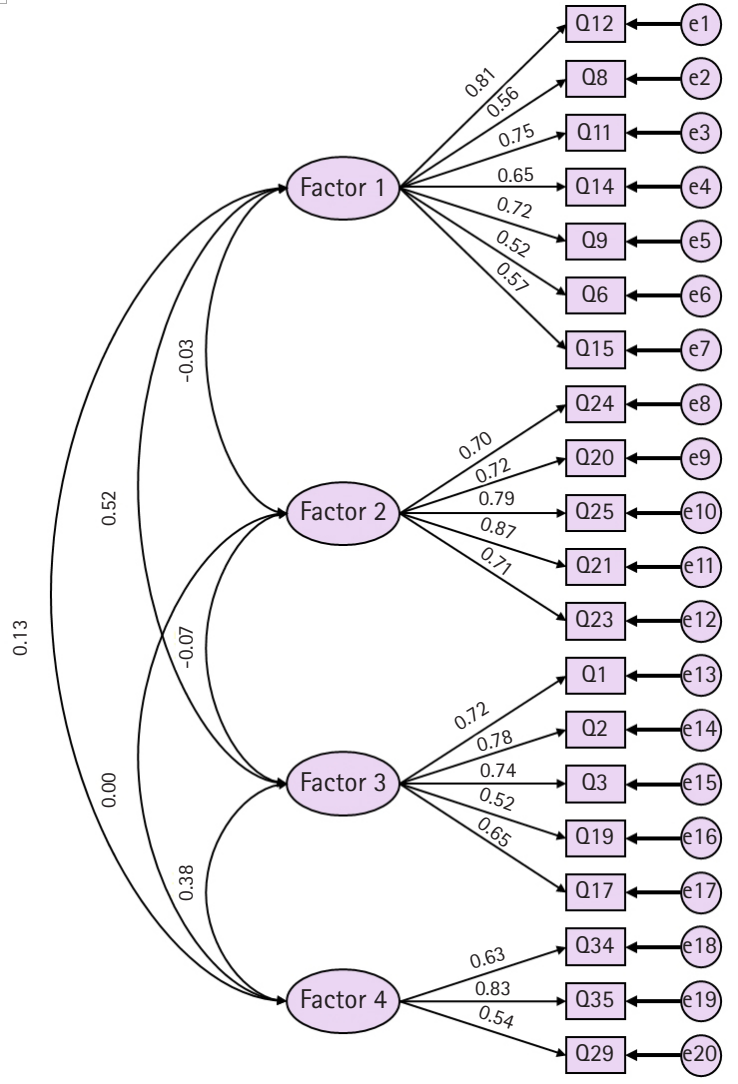
Model 3 with factor loadings for the 20-item Nursing Students’ Readiness for Flipped Classroom.

In addition, all items in factor 5 did not obtain an acceptable loading value. Therefore, it was decided to exclude factor 5 from model 3. Model 3 was found to be the model with the best fit in the current study (Fig. 1). According to SRMR and an adjunct discrepancy-based fit index (χ^2^/df), model 3 achieved an acceptable level of fit (SRMR=0.08 and χ^2^/df=1.57). In reference to RMSEA, model 3 demonstrated reasonable fit (RMSEA=0.08). The CFI of model 3 (0.87) came close to meeting the cut-off level (Table 3).

## Discussion

### Interpretation

Student readiness is recognized as a valuable factor for determining pedagogical effectiveness [4]. Therefore, the present study investigated the development and psychometric properties of a scale used to measure nursing students’ readiness for the FC. More specifically, the study explored the construct validity and reliability of the NSR-FC.

The method used to construct the NSR-FC was similar to the procedure used for the development of student readiness scales in other disciplines, and included proposing constructs, item generation, analysis of the content, item reduction, and validation of the newly developed instrument [6,7]. PCA revealed a potential 5-factor structure for the NSR-FC. However, CFA was only able to confirm a 4-factor model as determined by an inspection of the fit indices. The 4 factors that best fit the data with respect to the NSR-FC included technological readiness, environmental readiness, personal readiness, and pedagogical readiness.

The 4 best-fitting factors of the NSR-FC were comparable with existing inventories used to measure students’ readiness for blended learning in school-level education. Technological readiness is a common subset, identified as technology self-efficacy in many other studies [6,18]. Technological readiness denotes an individual’s willingness to leverage novel technologies for carrying out tasks [19]. Items relating to environmental readiness in the NSR-FC were seen to load together with the technological factor in the E-Learning Readiness Scale [7]. Personal readiness was recognized as a factor in the NSR-FC. Since FC is a student-centred pedagogical approach, students play a significant role in teaching-learning activities. Therefore, it is necessary to estimate students’ individual willingness to engage in the FC. Personal readiness was identified as “learner control,” with some variation, in the Online Learning Readiness Scale [6]. Pedagogical readiness in the NSR-FC describes students’ willingness to embrace learning content through FC pedagogy. A few items in the pedagogical readiness factor were correlated with the subscale of “online communication self-efficacy” in the Online Learning Readiness Scale [6] and the “content factor” in the E-Learning Readiness Scale [7].

In the reliability assessment, the NSR-FC demonstrated acceptable internal consistency. All 4 factors of the NSR-FC generated Cronbach α and ICC values greater than 0.7, confirming internal consistency within the domains and the ability of the NSR-FC to generate reproducible results. Therefore, the 4-factor model of NSR-FC is a valid and reliable tool that can be used to measure students’ readiness.

### Limitation/generalizability

This instrument can be used by nursing educators and curriculum planners to evaluate the effectiveness of FC pedagogy. However, the results of factor analysis may have been sample-specific, and the generalizability of these results is subject to the similarity of respondents to the sample. Since the study was conducted in Sri Lanka, the NSR-FC could be used to assess nursing students’ readiness for the FC in the context of South Asia. Moreover, the CFI value of the NSR-FC did not meet the acceptable level. This may have been due to the limited sample size and is acknowledged as a limitation of the study. Thus, future research is encouraged to perform CFA of the NSR-FC with a larger sample size. Lastly, the study was conducted through a self-reported questionnaire survey, which may have resulted in reporting biases such as social desirability bias [20]. Qualitative research will likely be instructive in terms of enabling a deeper understanding of the phenomena underlying nursing students’ readiness for the FC.

### Conclusion

These findings indicate that the NSR-FC is an acceptable instrument for measuring nursing students’ readiness for the FC in reference to its construct validity and reliability within the Sri Lankan nursing education context. The 4 readiness subscales were found to be technological readiness, environmental readiness, personal readiness, and pedagogical readiness. This finding may provide a good platform and frame of reference for nursing curriculum researchers and educational designers regarding the necessity of assessing students’ readiness for gaining actual educational achievements through the FC.

## Figures and Tables

**Table 1.** Results of principal component analysis with varimax rotation

Item	Factors				
	1	2	3	4	5
Q12^a)^	0.706				
Q8^a)^	0.696				
Q11^a)^	0.695				
Q14^a)^	0.676				
Q10	0.674				
Q13	0.664				
Q9^a)^	0.656				
Q7	0.631				
Q6^a)^	0.550				
Q15^a)^	0.511				
Q18	0.476				
Q16	0.476	0.474			
Q24^a)^		0.901			
Q20^a)^		0.896			
Q25^a)^		0.885			
Q21^a)^		0.861			
Q23^a)^		0.792			
Q1^a)^			0.790		
Q2^a)^			0.769		
Q3^a)^			0.672		
Q4			0.604		
Q19^a)^			0.438		
Q17^a)^			0.419		
Q28				0.741	
Q27				0.727	
Q26				0.721	
Q34^a)^				0.637	
Q35^a)^				0.630	
Q29^a)^				0.506	
Q22				0.437	
Q31					0.853
Q30					0.809
Q32			0.425		0.525
Q33			0.435		0.475
Eigenvalue	8.89	4.38	2.81	1.75	1.50
% of variance	25.42	12.51	8.02	5.01	4.29
% of cumulated variance	25.42	37.93	45.94	50.96	55.25
No. of items	12	5	6	7	4
Correlation (r) factor-total score	0.67	0.92	0.13	0.26	0.12

a) Items that were retained in the confirmatory factor analysis.

**Table 2.** Results of internal consistency

Factor no.	Factor name	Items	Cronbach α	ICC
1	Technological readiness	12	0.88	0.87^**^
2	Environmental readiness	5	0.93	0.92^**^
3	Personal readiness	6	0.76	0.76^**^
4	Pedagogical readiness	7	0.81	0.81^**^
5	Interpersonal readiness	4	0.78	0.77^**^
Overall		34	0.9	0.90^**^

ICC, intraclass correlation coefficient.

** P<0.01.

**Table 3.** Goodness-of-fit indices from confirmatory factor analysis to test the suitability of the Nursing Students’ Readiness for Flipped Classroom

Model	Model description	SRMR	CFI	RMSEA	χ^2^/df
1	Model with 34 items (after excluding item 5)	0.11	0.70	0.09	1.67
2	Model with item which obtained loading value >0.4	0.10	0.77	0.09	1.68
3	Model with 4 factors (after excluding factor 5)	0.08	0.87	0.08	1.57

SRMR, standardized root mean square residual; CFI, comparative fit index; RMSEA, root mean square error of approximation; df, degrees of freedom.

## Footnotes

**Data availability** Data files are available from Harvard Dataverse: https://doi.org/10.7910/DVN/IKRPCP Dataset 1. Two-hundred sixty-five students’ responses to the measurement scale of nursing students’ readiness for the flipped classroom for exploratory factor analysis and reliability testing. Dataset 2. Ninety students’ responses to the measurement scale of nursing students’ readiness for the flipped classroom for confirmatory factor analysis.

## Supplementary materials

Data files are available from Harvard Dataverse: https://doi.org/10.7910/DVN/IKRPCP

Supplement 1. Results of the content validation of the measurement scale of nursing students’ readiness for the flipped classroom.

Supplement 2. Audio recording of the abstract.
